# Why Do Empirical Valence Bond Simulations Yield Accurate Arrhenius Plots?

**DOI:** 10.1021/acs.jctc.4c00126

**Published:** 2024-03-07

**Authors:** Gabriel Oanca, Johan Åqvist

**Affiliations:** Department of Cell and Molecular Biology, Uppsala University, Biomedical Center, SE-751 24 Uppsala, Sweden

## Abstract

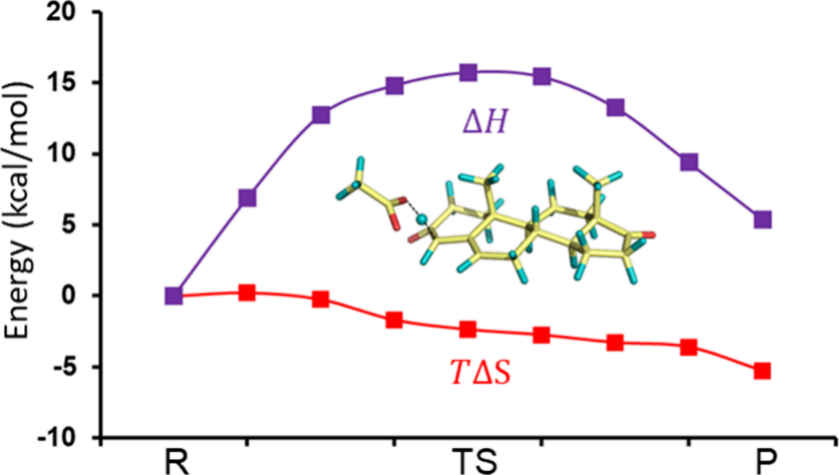

Computer simulations of the temperature dependence of enzyme reactions using the empirical valence bond (EVB) method have proven to give very accurate results in terms of the thermodynamic activation parameters. Here, we analyze the reasons for why such simulations are able to correctly capture activation enthalpies and entropies and how sensitive these quantities are to parametrization of the reactive potential energy function. We examine first the solution reference reaction for the enzyme ketosteroid isomerase, which corresponds to the acetate catalyzed deprotonation of the steroid in water. The experimentally determined activation parameters for this reaction turn out to be remarkably well reproduced by the calculations. By modifying the EVB potential so that the activation and reaction free energies become significantly shifted, we show that the activation entropy is basically invariant to such changes and that Δ*S*^⧧^ is instead determined by the specific mixture of the underlying force fields in the transition state region. The coefficients of this mixture do not change appreciably when the EVB potential is modified within reasonable limits, and hence, the estimate of Δ*S*^⧧^ becomes very robust. This is further verified by examining a more complex concerted hydride and proton transfer reaction in the enzyme hydroxybutyrate dehydrogenase.

## Introduction

The empirical valence bond (EVB) method^1,2^ has proven to be very useful for calculating thermodynamic activation parameters for chemical reactions, both in solution and in enzymes.^3−7^ In 2008, we devised a procedure analogous to experimental Arrhenius plots to obtain activation free energies as a function of temperature from molecular dynamics (MD) simulations in combination with the EVB method.^8^ With high enough precision for the calculated activation free energies, computational Arrhenius plots of either Δ*G*^⧧^/*T* vs 1/*T* or Δ*G*^⧧^ vs *T* can thus be constructed, which allows the values of Δ*H*^⧧^ and Δ*S*^⧧^ to be reliably extracted by linear regression. Today, this precision can typically be pushed to 0.1–0.2 kcal/mol, with many replicate MD/EVB free energy simulations, and the accuracy of the predicted Δ*H*^⧧^ and Δ*S*^⧧^ values is rather determined by how well the Arrhenius plot fits a straight line, than by the precision of the Δ*G*^⧧^ values. For a number of examined enzyme reactions, we found that the calculated thermodynamic activation parameters are in remarkably good agreement with experimental measurements. Some recent examples of this are cytidine deaminase,^9^ EF-Tu catalyzed GTP hydrolysis on the ribosome,^10^ α-amylase,^4^ hydroxybutyrate dehydrogenase,^5^ lactate dehydrogenase,^6^ and chorismate mutase.^7^

The most recent case of chorismate mutase is particularly instructive, since it is a unimolecular reaction in the enzyme (and in solution), with no enzymic groups participating in the actual chemistry. In that case, the EVB potential was parametrized on density functional theory (DFT) calculations on the uncatalyzed reaction in solution, with a few explicit water molecules together with a continuum solvent model.^7^ Precisely since the reaction is unimolecular, no thermodynamic corrections to the free energy profile in water, associated with bringing the reactants together in the same solvent cage, are required. The perfectly linear Arrhenius plots obtained from MD/EVB simulations of both the water reaction and the catalyzed reactions by enzymes from two different species turn out to give very accurate values of both activation enthalpies and entropies compared to experimental results.^7^

The question we want to address in this work is what is the underlying reason for this methodology giving such good estimates of the thermodynamic activation parameters? Here, it should, of course, be kept in mind that any enzyme EVB model has the value of Δ*G*^⧧^ somehow parametrized but, notably, not Δ*H*^⧧^ and Δ*S*^⧧^. This parametrization was initially most often performed against experimentally derived data for an uncatalyzed reference reaction in water.^1,2^ More lately, and for reference reactions lacking experimental data, the water reaction has been parametrized based on quantum chemical calculations with a suitable representation of the surrounding solvent.^3,4,7^ In both of these cases, it should be noted that no parametrization against enzyme data has been done, and the catalytic effect of “moving” the reference reaction into an enzyme is entirely a predicted result of the MD/EVB free energy calculations. An alternative that we have used recently for cases where the reference reaction would be rather complex is to parametrize the EVB potential energy surface directly on DFT calculations for the enzyme reaction. This can be done using either large cluster models of the active site with a continuum solvent model^9^ or using QM/MM calculations with explicit solvation.^5,11^ In order for such approaches to be viable, it is, of course, necessary that the DFT calculations give energetics that reasonably well match experimental results. Among the examples given above, we have representatives of all four variants of EVB parametrization (solution experiments,^10^ solution DFT,^3,4,7^ enzyme cluster DFT,^9^ and enzyme QM/MM DFT)^5,6^ and the predicted values of Δ*H*^⧧^ and Δ*S*^⧧^ are in all cases remarkably good.

## Theoretical Background

### EVB Methodology

To understand the origin of the thermodynamic activation parameters in the EVB model, it is useful to first review its basic features. An elementary chemical reaction step is usually treated as a two-state problem where the system is then represented by a 2 × 2 Hamiltonian matrix
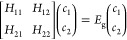
1Here, **c** is the eigenvector and the ground-state energy (*E*_g_) is obtained as the lowest eigenvalue of the secular equation pertaining to the above Hamiltonian:
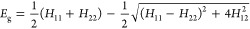
2where the diagonal matrix elements *H*_11_ and *H*_22_ are classical force fields representing the reactant and product states of the reaction step. The off-diagonal element *H*_12_ = *H*_21_ represents the coupling or mixing of the two states and is often taken as a constant term, although it can also be given a distance or energy gap dependent form.^1,2,12^ As said, *H*_11_ and *H*_22_ are standard force fields of the type
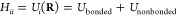
3where the bonded terms (*U*_bonded_) consist of bond (Morse potentials), angle and torsion terms, while the nonbonded terms (*U*_nonbonded_) denote electrostatic and van der Waals (Lennard-Jones) interactions as usual.

There is, however, something missing here. Consider for example the simple proton transfer between two water molecules, H_2_O + H_2_O ⇌ OH^–^ + H_3_O^+^. If we keep the two molecules on each side of the equation at their equilibrium geometries and at infinite separation from each other (meaning no nonbonded interactions between them), then the force field energies would be *U*_1_(**R**) = *U*_2_(**R**) = 0. This is clearly not correct since there is an absolute energy difference between the left- and right-hand sides of the equation under such a condition. That is, there is a real difference in the heat of formation between 2H_2_O and OH^–^ + H_3_O^+^ that is not accounted for by standard force fields. Hence, we must include such a constant term, and this is, e.g., done by modifying *U*_2_(**R**) to *U*_2_(**R**) + Δα, where Δα is formally the absolute energy difference between the reactant and product when the reacting groups are at their equilibrium geometries and noninteracting.

**Figure 1:**
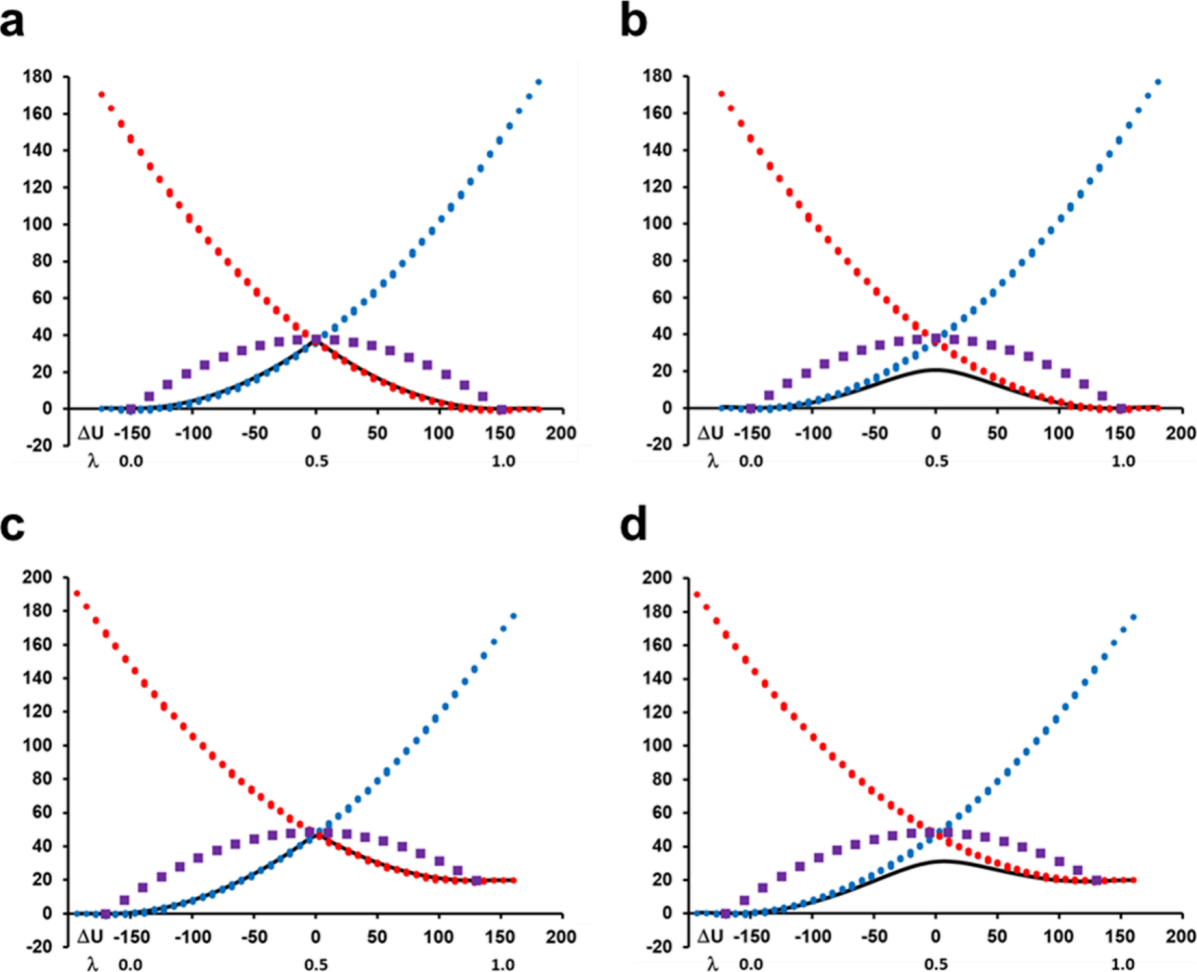
Effect of the EVB parameters *H*_12_ and Δα on the free energy profiles for transfer of a negative charge between Na and Na^+^ in water at a 6 Å separation. The curves with purple squares denote the mapping free energy Δ*G*(λ_m_). Red and blue circles are the diabatic (Δ*G*_1_ and Δ*G*_2_) free energy curves, and the solid black curves denote the ground-state free energy (Δ*G*_*g*_). The following sets of *H*_12_ and Δα parameters are used: (a) 0, 0; (b) 20, 0; (c) 0, 20; and (d) 20, 20. The quantities Δ*G*_1_, Δ*G*_2_, and Δ*G*_*g*_ are functions of energy gap Δ*U*.

We now thus have two unknown parameters in our EVB model that are not given by any force field, namely, *H*_12_ and Δα. This is also the beauty of the method, that these two parameters can be used to calibrate the energetics of the EVB model so that it reproduces whatever data are used to parametrize the model. In practice, it is the activation free energy Δ*G*^⧧^ and the reaction free energy Δ*G*^0^ that are parametrized by parameters *H*_12_ and Δα. To clarify the concepts a bit further, let us consider an even simpler test case, the transfer of an electronic charge between a sodium atom and a sodium ion at fixed distance in water (Na + Na^+^ ⇌ Na^+^ + Na), with *H*_12_ = 0 and Δα = 0. This is thus just a hypothetical transformation without consideration of electron tunnelling or anything like that. We use the free energy perturbation (FEP) method to map the free energy change upon moving from reactant to product, via a mapping potential expressed as a linear combination of the two end-states:
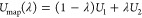
4The free energy of the system for any value of the mapping parameter λ_m_ relative to the reactant state (λ = 0) is given by summation of Zwanzig’s formula:^13^
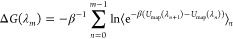
5where λ has now been discretized into a number of points λ_*n*_ (or windows) and the MD ensemble average at each such point estimates the free energy change associated with moving to the next point. The free energy change as a function of λ going from 0 to 1 is shown in Figure 1a together with the free energy change on the ground-state potential given by eq 2.

This ground-state free energy profile is obtained with the umbrella sampling formula from the free energy on the mapping potential Δ*G*(λ_m_) according to^1,2,12^
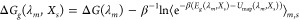
6where the generalized reaction coordinate is the energy gap occurring in eq 2, *X*_s_ = Δ*U* = *U*_1_ – *U*_2_. Since each value of λ_m_ only samples a certain range of the energy gap, the overall free energy profile is obtained by piecing together these ranges according to^12^
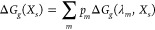
7where the sum runs over all the sampling windows (λ_m_) that contribute to the bin *X*_*s*_ and *p*_*m*_ is the normalized statistical weight of the *m*^th^ sampling window in that bin. This gives the solid black ground-state free energy curve shown in Figure 1a, with a free energy barrier of Δ*G*^‡^ = 37 kcal/mol that is entirely due to solvent reorganization. Likewise, the free energy functions corresponding to the pure reactant and product states can be obtained by the equivalent of eq 6 where *E*_g_ is replaced by either *U*_1_(**R**) or *U*_2_(**R**)
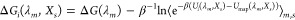
8where *i* = 1 and 2 and pieced together again as before
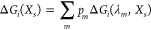
9These diabatic free energy curves are shown with blue and red circles in Figure 1a. As can be seen, Δ*G*_1_ and Δ*G*_2_ intersect at precisely 37 kcal/mol, and this is also the value of Δ*G*(λ_*m*_) at λ = 0.5. Now, what happens if we introduce a coupling between the two states in terms of a nonzero value of *H*_12_? The result of introducing such a coupling for our Na + Na^+^ ⇌ Na^+^ + Na test case with *H*_12_ = 20 kcal/mol is shown in Figure 1b. As can be seen, neither Δ*G*(λ_*m*_) nor the diabatic Δ*G*_*i*_’s have changed, but the adiabatic ground-state free energy function now has a lower barrier of Δ*G*^‡^ = 20 kcal/mol due to the coupling between reactant and product states, while Δ*G*^0^ is still zero. If we instead keep *H*_12_ = 0 and change our Δα from zero to 20 kcal/mol, all that happens to our free energy curves is that the product state is lifted by 20 kcal/mol as can be seen from Δ*G*(λ_*m*_) and Δ*G*_2_ in Figure 1c, and the reaction free energy thus becomes Δ*G*^0^ = 20 kcal/mol. As the diabatic free energy curve of the product state becomes shifted upward, the intersection between Δ*G*_1_ and Δ*G*_2_ will also be up-shifted, and the barrier on Δ*G*_*g*_ now becomes 47 kcal/mol. Finally, to illustrate how the EVB potential can be parametrized to any target values of Δ*G*^⧧^ and Δ*G*^0^, Figure 1d shows the effect of setting the parameters to *H*_12_ = 20 kcal/mol and Δα = 20 kcal/mol. Now we get Δ*G*^‡^ = 31 kcal/mol and Δ*G*^0^ = 19 kcal/mol on the ground-state free energy curve.

**Figure 2:**
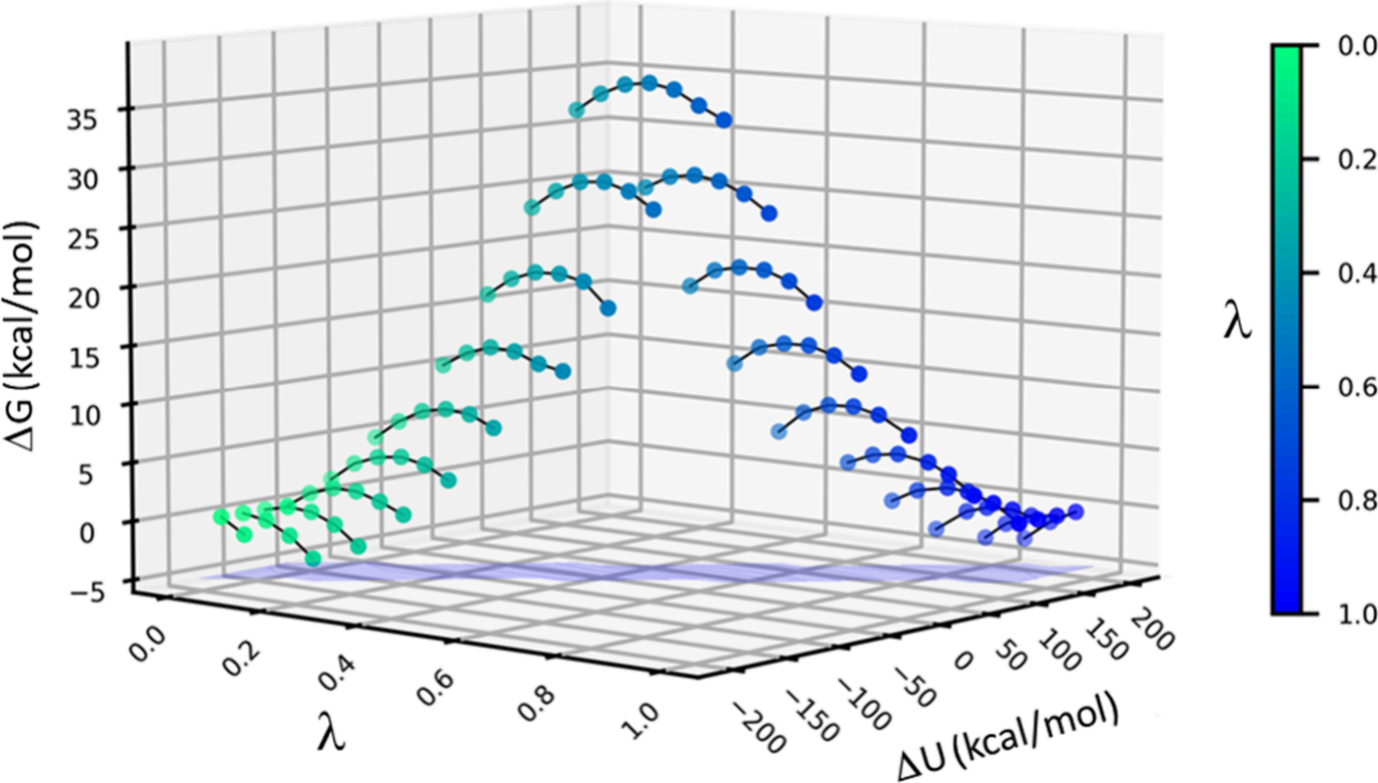
Illustration of how eqs 6 and 7 work in practice. Each value of λ (color coded form green to blue) contributes to several bins of the discretized energy gap Δ*U* (denoted by solid lines). The resulting free energy value of each bin is obtained by statistical weighting of the contributions from each λ.

The calculation of the ground-state free energy profile according to eqs 6 and 7 can be further illustrated graphically, as shown in Figure 2, where it can be seen how different values of the mapping parameter λ will sample different regions of the energy gap Δ*U*. It should be noted here that all the dynamics used for sampling the system are run on the artificial potential *U*_map_(λ). However, with a parametrized EVB model at hand, it is straightforward to also run the dynamics on the actual ground-state potential. This may be of interest for studying the true dynamic properties of the reacting system, for example, for evaluating the transmission coefficient in transition state theory. The forces acting on the particles of the system will then depend on the energy gap and the off-diagonal matrix element according to the derivatives:
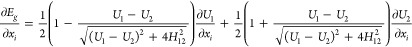
10where *x*_*i*_ is any given Cartesian coordinate, and *H*_12_ is assumed to be a constant for simplicity (with a distance or energy gap dependent *H*_12_, the expression becomes slightly more complicated).

### Computational Arrhenius Plots from MD/EVB Simulations

**Figure 3:**
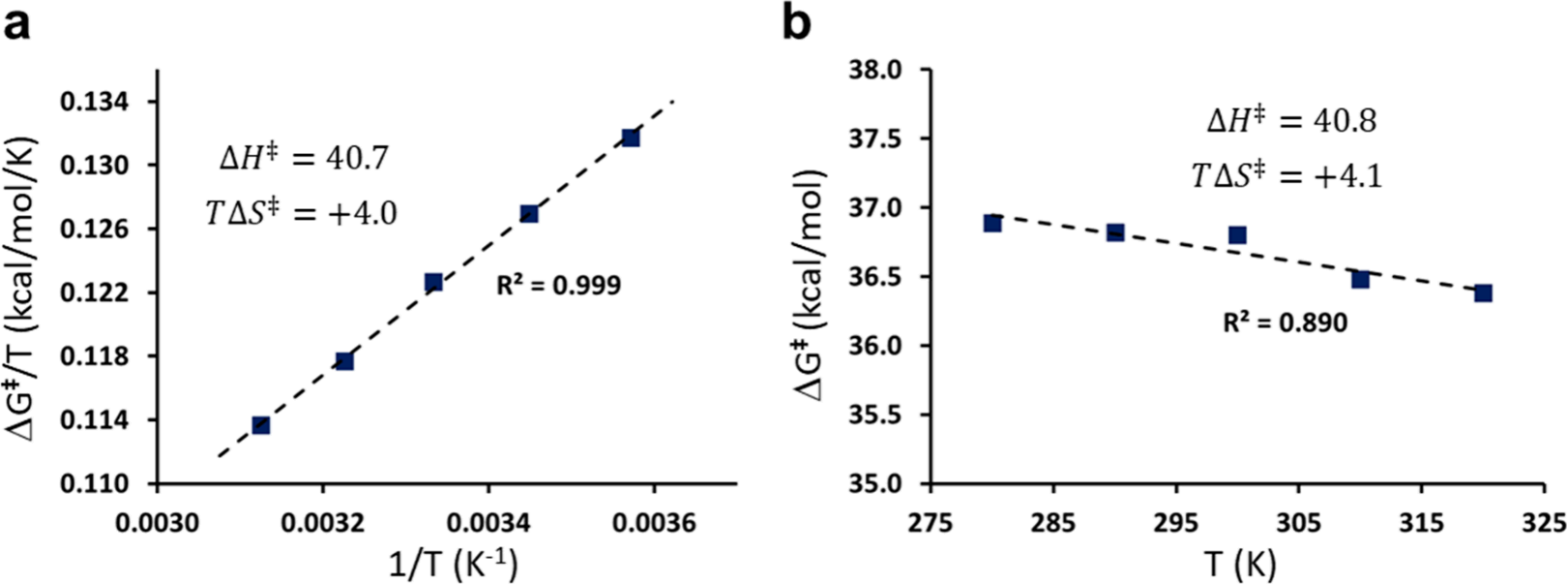
Calculated Arrhenius plots of (a) Δ*G*^⧧^/*T* vs 1/*T* and (b) Δ*G*^⧧^ vs *T* for the transfer of a negative charge between Na and Na^+^ in water at 6 Å separation with *H*_12_ = 0.

As an example of how the enthalpy and entropy components of the activation free energy are extracted from MD/EVB simulations, let us again consider the simple Na + Na^+^ ⇌ Na^+^ + Na test case with *H*_12_ = 0 and Δα = 0. We thus repeat the free energy calculations at a set of different temperatures, e.g., in the range 280–320 K in steps of 10 K, and plot Δ*G*^⧧^/*T* vs 1/*T* or, alternatively, Δ*G*^⧧^ vs *T*. Depending on the magnitude of Δ*H*^⧧^ relative to Δ*S*^⧧^ the linear regression will be more accurate with a higher *R*^2^ value for either of the two types of plots. That is, for a reaction with a high enthalpy component, the Δ*G*^⧧^/*T* vs 1/*T* plot will be the most accurate since the enthalpy then corresponds to the slope of the plot, as opposed to the Δ*G*^⧧^ vs *T* variant where the entropy becomes the slope. This is illustrated for our test case in Figure 3, where it can be seen that Δ*H*^‡^ = 40.7 kcal/mol and Δ*S*^‡^ = 0.0134 kcal/mol/K, corresponding to *T*Δ*S*^‡^ = +4.02 kcal/mol at 300 K. Hence, the free energy barrier for transfer of an electronic charge from Na to Na^+^ is completely dominated by the enthalpy barrier, while the entropy contribution is favorable at the transition state. This can be understood in terms of the strong electrostatic interactions between water and a solvated ion that partly have to break in the transition state region, where the positive charge becomes delocalized between the two Na atoms. As a solvated alkali ion also has an unfavorable entropy contribution to the solvation free energy of about *T*Δ*S*_sol_ = −6 kcal/mol,^14^ the delocalization of the charge reduces this entropy penalty, due to weaker ion–water interactions, and the contribution to the reaction barrier (−*T*Δ*S*^‡^ < 0) thus becomes favorable.

Below we will examine the performance of this approach for calculating thermodynamic activation parameters for a number of test cases, including reactions both in solution and in eznymes. We are particularly interested in assessing the influence of EVB parameters *H*_12_ and Δα on the enthalpy–entropy balance of the activation free energy. Our analysis demonstrates that the EVB parameters, together with the target value of Δ*G*^⧧^, largely dictate the activation enthalpy, while the entropic contribution is rather intrinsic to the force field. That is, the entropy change upon moving from the reactants to the particular mixture of *U*_1_ and *U*_2_ representing the transition state is found to be almost invariant, with respect to the EVB parameters.

## Results

### Simulations of the Water Reference Reaction for Ketosteroid Isomerase

**Figure 4:**
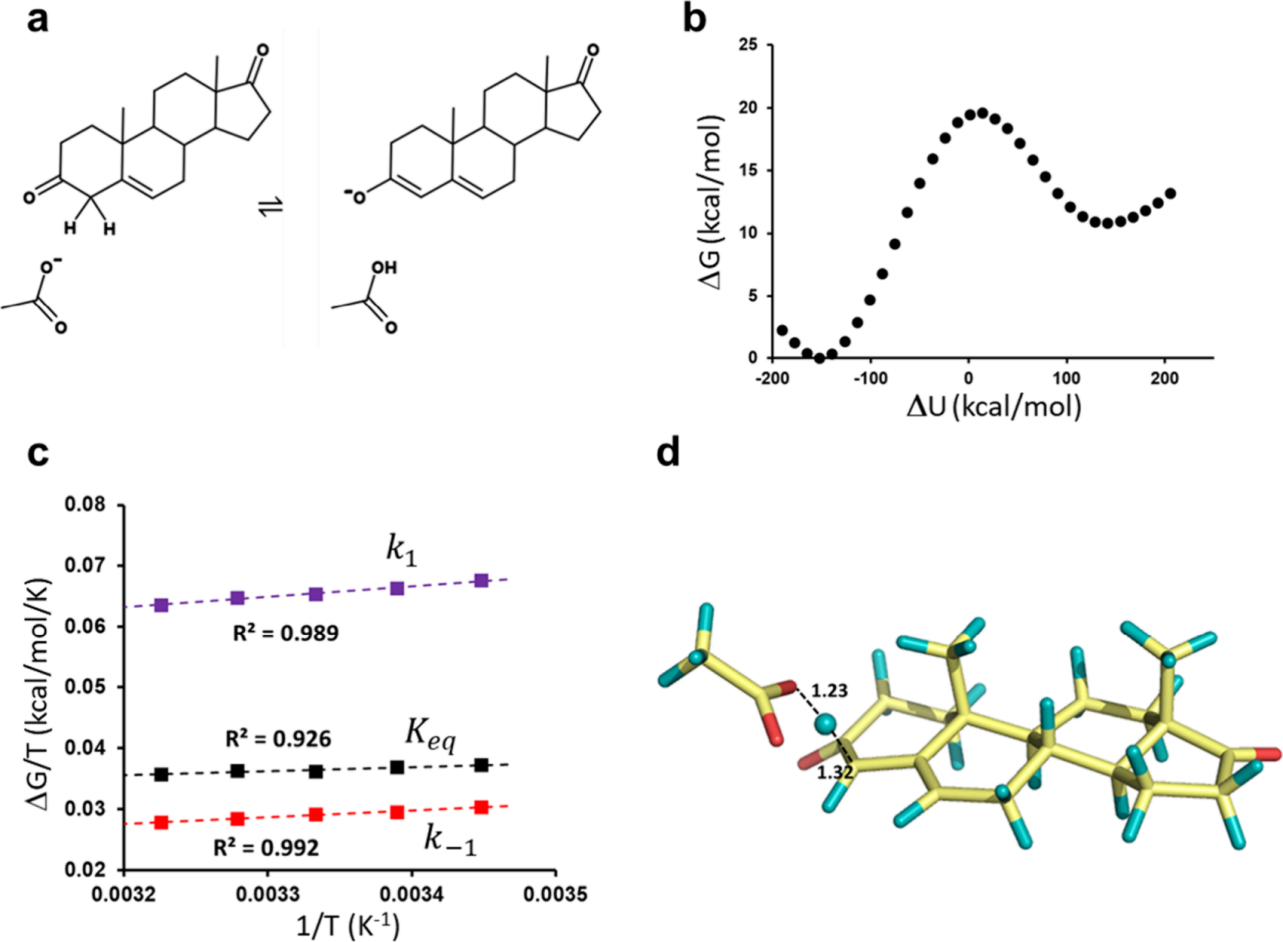
(a) Mechanism of the acetate catalyzed proton abstraction from the 5-androstene-3,17-dione steroid. (b) Free energy profile of the reaction obtained from MD/EVB simulations. (c) Calculated Arrhenius plots from the forward (*k*_1_) and backward (*k*_–1_) reactions and for the equilibrium constant (*K*_eq_). (d) MD snapshot from the transition state region of the reaction.

As an example of a relatively simple solution reaction, we choose the acetate catalyzed proton abstraction from 5-androstene-3,17-dione (Figure 4a). This is the solution reference for the first step of the ketosteroid isomerase reaction, where an aspartate residue abstracts the 4β proton from the steroid substrate. Houck and Pollack have determined the activation parameters for this reaction in water both in the forward and backward directions.^15,16^ We thus simulate this reaction in a 40 Å diameter water sphere with the MD/EVB methodology described above and the standard OPLS-AA/M force field,^17^ where the experimentally determined values of Δ*G*^⧧^ = 22.0 and Δ*G*^0^ = 10.9 kcal/mol at 298 K are used as calibration target values for the EVB model. The parametrization against experimental data also means that any nuclear (proton) tunnelling effects are implicitly included in the effective EVB potential.^18^

Here, however, we encounter the typical problem of simulating bimolecular reactions in solution where the experimental standard state is taken as 1 M. In microscopic terms, reaction rates with this standard state also include the probability of an encounter between the donor and acceptor atoms in addition to the reaction rate for the contact complex. If we set our origin at one of the reacting partners, a 1 M concentration of the other reactant around it means that it is free to move within 1661 Å^3^, or within a radius of about 7.3 Å. Hence, for the encounter probability to be accurately treated, it would be necessary for the MD simulation at the reactant state to correctly sample the diffusion within this 1661 Å^3^ sphere. Since such a calculation of what is mostly entropy is known to converge very slowly,^19^ one instead usually employs a formal correction to the 1 M reaction rates. That is, a distance restraint that keeps the donor and acceptor in contact with each other is used, and the formal concentration of the otherwise diffusing partner is then considered to be the same as the surrounding solvent. For a reaction in water, this means that the concentration of the diffusing reactant has changed from 1 to 55 M, implying that the measured activation free energy should be corrected by −*RT* ln(55) = −2.39 kcal/mol at room temperature. With this translational entropy correction, our target value for Δ*G*^⧧^ becomes 19.6 kcal/mol at a 55 M standard state, while Δ*G*^0^ is unaffected since the equilibrium constant between reactant and product does not depend on the standard state. A typical calculated reaction free energy profile at 300 K is shown in Figure 4b.

The results from these calculations of acetate catalyzed deprotonation of 5-androstene-3,17-dione at five different temperatures (290, 295, 300, 305, and 310 K) are shown in the Arrhenius plots of Figure 4c and a typical MD snapshot from the transition state region is shown in Figure 4d. At each temperature, 60 independent reaction free energy profiles were calculated, and each such profile corresponds to 1.05 ns of MD simulation, where 21 distinct λ windows (eq 4) were used. As can be seen, the Arrhenius plots are highly linear with *R*^2^ values above 0.93, and the extracted thermodynamic activation parameters are summarized in Table 1. It is immediately apparent that the enthalpies and entropies for both forward and backward reactions as well as for the equilibrium constant are reproduced by the MD/EVB simulations to within the combined simulation and experimental errors. Particularly, the fact that the entropy terms become correct clearly supports the approximation above, where the change from a 1 to a 55 M standard state is considered solely as a translational entropy effect. That the free energies are exactly reproduced is obvious in view of the EVB parametrization, but the agreement with regard to the enthalpy and entropy components is quite remarkable since only their sum is subject to fitting.

**Table 1:** Calculated and Experimental^15,16^ Thermodynamic Parameters for the Acetate Catalyzed Proton Transfer Reaction in Water at 300 K (kcal/mol)^a^

reaction	Δ*G*_calcd_^⧧^	Δ*G*_expt_^⧧^	Δ*H*_calcd_^⧧^	Δ*H*_expt_^⧧^	*T*Δ*S*_calcd_^⧧^	*T*Δ*S*_expt_^⧧^
*k*_1_	19.6 ± 0.04	19.6 ± 0.1	17.3 ± 1.0	16.4 ± 1.9	–2.3 ± 1.0	–2.6 ± 1.8
*k*_–1_	8.7 ± 0.10	8.7 ± 0.1	11.0 ± 0.6	9.6 ± 1.8	2.3 ± 0.6	1.7 ± 0.3

	Δ*G*_calcd_^0^	Δ*G*_expt_^0^	Δ*H*_calcd_^0^	Δ*H*_expt_^0^	*T*Δ*S*_calcd_^0^	*T*Δ*S*_expt_^0^
*K*_eq_	10.9 ± 0.09	10.9 ± 0.1	6.4 ± 1.0	6.8 ± 1.9	–4.6 ± 1.0	–4.3 ± 1.8

a The experimental activation free energies and entropies have been corrected to represent a 55 M standard state, as described in the text. Error bars for the calculated free energies denote the standard error of the mean (s.e.m.) at 300 K from 60 replicate simulations, while those for enthalpies and entropies are the asymptotic standard errors from linear regression.

To shed some light on the origin of the enthalpy and entropy terms, it is useful to explore the effect of modifying the EVB potential by varying *H*_12_ and Δα. That is, by changing these parameters, but using exactly the same underlying MD trajectories, we can move the diabatic free energy curves and the transition state on the ground-state potential up or down, as shown in Figure 1. Starting from our standard EVB parametrization for the proton transfer reaction, with *H*_12_ = 52.6 and Δα = 99.55 kcal/mol, we add or subtract 10 kcal/mol to each of these parameters and recalculate the Arrhenius plots for the forward reaction. The results of this exercise are summarized in Table 2, where it can be seen that Δ*G*^⧧^ goes up when Δα is increased and when *H*_12_ is decreased and down when these parameters are changed in the opposite directions. This thus follows the logic of Figure 1. However, what is interesting here is that it is Δ*H*^⧧^ that closely follows the shifts in Δ*G*^⧧^ while *T*Δ*S*^⧧^ is almost invariant with our modifications of the EVB potential. Hence, the *T*Δ*S*^⧧^ term must be intrinsically determined by the underlying force fields *U*_1_(**R**) and *U*_2_(**R**), OPLS-AA/M in our case.^17^

**Table 2:** Effect of Modifying *H*_12_ and Δα on the Activation Parameters for the Forward Proton Transfer Reaction at 300 K (kcal/mol)^a^

*H*_12_	Δα	Δ*G*_calcd_^⧧^	Δ*H*_calcd_^⧧^	*T*Δ*S*_calcd_^⧧^
52.6	99.55	19.6	17.3	–2.3
52.6	109.55	24.4	22.2	–2.3
52.6	89.55	15.1	13.0	–2.1
62.6	99.55	15.8	13.4	–2.4
42.6	99.55	24.3	22.1	–2.2

a The standard EVB parametrization corresponds to the first row in the table.

Since the *T*Δ*S*^⧧^ term is found to be invariant with respect to the EVB parametrization for our proton transfer reaction, this must mean that the geometry and charge distribution of the transition state region does not change appreciably when we modify *H*_12_ and Δα as above. This would imply that the state coefficients, *c*_1_^2^ + *c*_2_^2^ = 1, of the ground-state eigenvector at the transition state do not change very much when we modify the EVB potential. These coefficients are given by
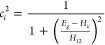
11and the average values of *c*_1_^2^ and *c*_2_^2^ for our standard parametrization are 0.45 and 0.55 at the transition state, respectively. As an example, when we change Δα to 89.55 kcal/mol (third entry in Table 2), these coefficients change only to 0.47 and 0.53. This means that the transition state region will largely be sampled by the same λ windows, and hence, its entropy relative to the reactant state is determined by the corresponding mixture of *U*_1_ and *U*_2_. This can be further verified by simply calculating the Arrhenius plot corresponding to the free energy difference between Δ*G*(λ_*m*_) at λ = 0.55 and λ = 0.10. This free energy difference thus has nothing to do with EVB but only represents the change of mapping potential from the reactant minimum in Figure 4b (λ = 0.10) to the mixture of *U*_1_ and *U*_2_ that roughly corresponds to the transition state (λ = 0.55). While the enthalpy change between these two mapping points is much larger than Δ*H*^⧧^, since there is no *H*_12_ coupling involved in *U*_map_(λ) (Figure 1), it turns out that the corresponding *T*Δ*S*(λ = 0.55) – *T*Δ*S*(λ = 0.10) = −2.6 kcal/mol, which is very close to the value of *T*Δ*S*^⧧^ obtained in Table 2. Hence, we can conclude that the entropy term at the transition state is largely determined simply by a mixture of the underlying state potentials.

**Figure 5:**
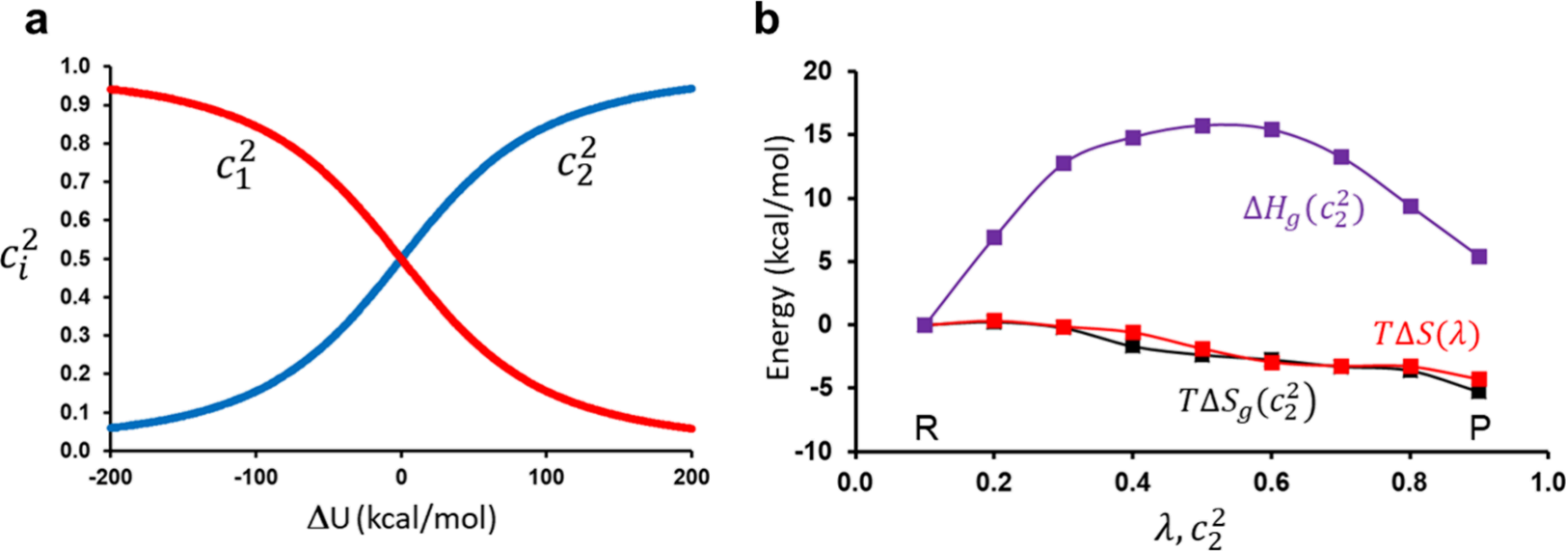
(a) Relationship between the energy gap and the EVB state coefficients corresponding to the reactant and product weights obtained from the MD/EVB simulations at 300 K. (b) Calculated thermodynamic parameters (Δ*H*_*g*_ and *T*Δ*S*_*g*_) along the ground-state reaction path from the reactant to the product minimum, together with the entropy term along the mapping potential (eq 4). Note that the corresponding enthalpy on the mapping potential (not shown) is considerably higher since it does not involve any coupling between the states. Data for all three curves are plotted relative to the reactant minimum.

The above conclusion can be further illustrated for our proton transfer reaction by comparing the *T*Δ*S* term along the mapping potential (eq 4) with that on the ground state along the reaction coordinate. In the latter case, we therefore bin the value of *c*_2_^2^ along the reaction coordinate Δ*U* to get data points that more closely correspond to the λ values than the Δ*U* coordinate does (Figure 5a). For each such bin, we then calculate an Arrhenius plot from the five temperatures, both for Δ*G*(λ) and for Δ*G*_*g*_(*c*_2_^2^), and the result is shown in Figure 5b in terms of *T*Δ*S*(λ), *T*Δ*S*_*g*_(*c*_2_^2^), and the ground-state enthalpy term Δ*H*_*g*_(*c*_2_^2^). The curves in Figure 5b are plotted from the reactant minimum at λ = *c*_2_^2^ = 0.10 to the product minimum at λ = *c*_2_^2^ = 0.90. It can be seen that *T*Δ*S*_*g*_(*c*_2_^2^) closely follows *T*Δ*S*(λ) with a value at the transition state (λ = *c*_2_^2^ = 0.55) of −2.5 kcal/mol (at 300 K). Moreover, the entropy term decreases monotonically along the path from reactant to product for this reaction, while the corresponding enthalpy term Δ*H*_*g*_(*c*_2_^2^) reaches a maximum at the transition state and then decreases toward the product.

### Simulations of Hydride Transfer in a Short Chain Dehydrogenase

**Figure 6:**
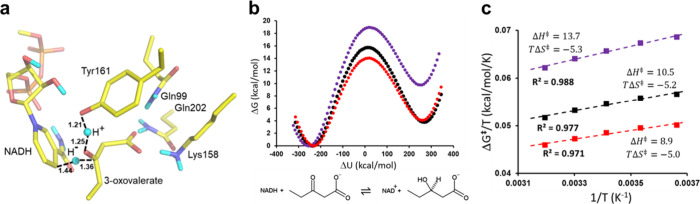
(a) View of the EVB transition state for concerted hydride and proton transfer in HBDH, where a typical MD snapshot at the top of the free energy barrier is shown. Calculated free energy profiles for the reaction with the standard *H*_12_ and Δα parameters (168.54 and 96.70 kcal/mol–black circles)^23^ and with 10 kcal/mol added to either *H*_12_ (red circles) or Δα (purple circles). (c) The corresponding Arrhenius plots from five different temperatures for the three cases of EVB parameters (color coding as in panel b).

In order to address the generality of the above findings, particularly the insensitivity of *T*Δ*S*^⧧^ to the EVB parametrization, we also examine the energetics of an enzyme reaction. Here, we will consider the reduction of 3-oxovalerate to (*R*)-3-hydroxyvalerate by the psychrophilic hydroxybutyrate dehydrogenase enzyme (HBDH) from *Psychrobacter arcticus*. This reaction proceeds by concerted hydride and proton transfer to the substrate from the cofactor NADH and Tyr161, respectively (Figure 6a).^11,20^ It is an example with a relatively large experimentally determined entropy penalty for the tetrameric enzyme of *T*Δ*S*^⧧^ = −6.1 kcal/mol at 283 K.^20^ We earlier modeled it via MD/EVB simulations using both the Q^21^ and GROMACS^22^ software for different oligomeric states of the enzyme.^5,23^ The tetramer calculations gave an entropy penalty of *T*Δ*S*_calcd_^⧧^ = −6.4 kcal/mol, in excellent agreement with experimental results, and interestingly, the thermodynamic activation parameters were found to be more or less insensitive to the oligomeric state.^5^ Further, the calculations using Q and GROMACS for the HBDH monomer gave virtually identical results (*T*Δ*S*_calcd_^⧧^ = −5.5 and *T*Δ*S*_calcd_^⧧^ = −5.2 kcal/mol, respectively, at 283 K),^5,23^ and here we will use the latter set of calculations as an example.

The EVB model for HBDH was parametrized previously on average minimum energy paths from 10 replicate QM/MM minimum energy paths with final energies calculated at the M062X/ma-def2-TZVPP level, including thermal corrections at 283 K.^11^ These calculations turned out to yield very accurate results compared to experimental kinetic data, and the predicted values of the activation and reaction free energies were Δ*G*^⧧^ = 15.9 and Δ*G*^0^ = +3.9 kcal/mol (Δ*G*_expt_^⧧^ = 16 kcal/mol).^11^ The EVB calibration from GROMACS simulations of the monomer reaction in a periodic dodecahedral box at 283 K gave parameter values of *H*_12_ = 168.54 and Δα = 96.70 kcal/mol and the resulting free energy profile is shown in Figure 6b.^23^ The corresponding Arrhenius plot obtained from these MD/EVB simulations at 273, 283, 293, 303, and 313 K is shown in Figure 6c, and it predicts values of Δ*H*^⧧^ = 10.5 and *T*Δ*S*^⧧^ = −5.2 kcal/mol at 283 K.

As above, we now examine the effect of changing the EVB parametrization by adding 10 kcal/mol to either *H*_12_ or Δα. Addition of this energy quantity to Δα basically lifts the product state of the reaction, and as expected, we now get an increase of both Δ*G*^⧧^ and Δ*G*^0^ to 19.1 and 9.9 kcal/mol, respectively (Figure 6b). The activation enthalpy increases from Δ*H*^⧧^ = 10.5 to Δ*H*^⧧^ = 13.7 kcal/mol and, hence, perfectly matches the corresponding shift in Δ*G*^⧧^ (Figure 6c). There is thus no significant change of the entropy penalty, *T*Δ*S*^⧧^ = −5.3 kcal/mol (at 283 K). Likewise, if we add 10 kcal/mol to *H*_12_, the main effect is that the free energy barrier goes down due to the increased coupling between reactant and product states, and we now get Δ*G*^⧧^ = 14.0 and Δ*G*^0^ = 4.1 kcal/mol (Figure 6b). Again, this shift is basically only enthalpic, and the activation parameters now become Δ*H*^⧧^ = 8.9 and *T*Δ*S*^⧧^ = −5.0 kcal/mol (Figure 6c). Hence, as with the case of our proton transfer reaction in solution, we can conclude that the activation entropy penalty is not really determined by the EVB parameters, but by the degree of mixing of the two states in the transition state region. In this case, we also have *c*_1_^2^ and *c*_2_^2^ both ∼0.5 (*c*_1_^2^ = 0.48, *c*_2_^2^ = 0.52), and the entropy penalty is thus mainly determined by this particular mixing of the reactant and product force fields. As before, the entropy term on the ground state basically follows that on the mapping potential, and both give entropy penalties of *T*Δ*S* ≈ – 5 kcal/mol at λ = *c*_2_^2^ = 0.52 (at 283 K).

## Discussion

Herein, we have addressed the question of why EVB simulations are able to produce such an accurate description of the temperature dependence of chemical reactions in enzymes and solution in terms of computational Arrhenius plots. Obviously, if the activation free energy is correctly predicted at a given temperature, this just means that the sum of Δ*H*^⧧^ and – *T*Δ*S*^⧧^ is correctly predicted. However, since these components of the free energy are never individually involved in the EVB parametrization, it appears somewhat surprising that they are also almost always correctly predicted.

We have shown here that the answer to this lies in the use of an accurate classical force field for the reactant and product states, in the case of a chemical reaction step that can be represented by a two-state EVB Hamiltonian. Moreover, for enzyme reactions that are parametrized on a reference reaction in solution and not on large DFT cluster or QM/MM enzyme models, the force field description must also capture the effect of changing the surroundings of the reacting groups from water to the enzyme environment. A case in point here is the recent MD/EVB study of chorismate mutase where, first, the uncatalyzed solution reaction at 298 K was parametrized on DFT calculations (in terms of Δ*G*^⧧^ and Δ*G*^0^) with a mixed explicit/implicit solvent representation.^7^ MD/EVB simulations of the temperature dependence then gave predictions of Δ*H*^⧧^ and −*T*Δ*S*^⧧^ within 0.4 kcal/mol of the experimental values for the solution reaction. Upon transfer of the solution EVB model to two different enzyme variants, simulations of the catalyzed reactions also gave excellent agreement with the experimentally derived thermodynamic parameters and, particularly so, for the *T*Δ*S*^⧧^ terms that were within 0.7 kcal/mol of the measured values.^7^

Our analysis herein shows that the entropy term is basically determined by the mixture of *U*_1_ and *U*_2_ that defines the transition state region. This mixture is typically near 50/50 proportions for reactions that are not extremely endothermic or exothermic (for an example of more complex three-state EVB simulations, see ref (24)). The acetate catalyzed abstraction of a proton from androstenedione is an example of this. The entropy change on the mapping potential *U*_map_(λ) between the λ values corresponding to the transition state and reactant minimum is thus almost the same as the entropy change on the EVB ground-state potential. Even for more complex chemical reactions that involve more bonds being simultaneously broken and formed, the correspondence between the entropy on the two potentials is close, and *T*Δ*S*_*g*_ remains essentially insensitive to the EVB parametrization. This is illustrated by the concerted HBDH reaction above, which involves four bonds being simultaneously broken or formed. Hence, we can conclude that an accurate force field, itself, will ensure a good estimate of the activation entropy penalty, irrespective of how the EVB model is parametrized. This means that if the EVB model gives an accurate prediction of Δ*G*^⧧^, then it will also give a good estimate of Δ*H*^⧧^ (since Δ*G*^⧧^ = Δ*H*^⧧^ – *T*Δ*S*^⧧^).

It may further be useful to point out here that, besides the *H*_12_ and Δα parameters, the EVB model also offers the possibility of more detailed geometric parametrization of the transition state. That is, a softer exponential repulsion term (*U*_rep_ = *A*e^–β*r*_*ij*_^) instead of the Lennard-Jones 1/*r*^12^ is usually used between atoms involved in bond formation and breaking, and this repulsion can be tuned to obtain desired distances.^23^ Moreover, coupling terms between the Morse potentials describing changes in bonding are also usually introduced with respect to bond angles, torsions, and improper dihedrals, so that such terms vanish as bonds dissociate.^25^ Taken together, the parametrization of Δ*G*^⧧^ in terms of *H*_12_ and Δα from suitable target data, possibly with additional fine-tuning of the transition state geometry, makes the EVB model very powerful for describing chemical reactions both in solution and in enzymes. What makes the method capable of correctly also describing the temperature dependence of reactions turns out to be the fact that the underlying force fields are accurate enough to properly estimate the entropy changes along the reaction path.

## Methods

### Simulation of Charge Transfer between Na and Na^+^

The Na atom and Na^+^ ion, described by standard parameters,^26^ were enclosed in a TIP3P water sphere of diameter 40 Å and kept fixed at a 6 Å separation, by restraining each ion to its initial position (by 100 kcal/mol/Å^2^). For this system, 10 replicate MD/FEP simulations (Δ*t* = 1 fs) were carried out at each temperature (280, 290, 300, 310, and 320 K), starting from different initial velocities. Each replica involved 1.25 ns of heating and equilibration at the target temperature, followed by a 1.05 ns FEP calculation where the charge was moved from Na^+^ to Na in 21 discrete λ steps (eq 4).

### Acetate Catalyzed Deprotonation of 5-Andro-3,17-dione

The reactants and products were described by the OPLS-AA/M force field,^17^ and parameters were generated with Schrödinger’s ffld_server.^28^ The reacting molecules (acetate and steroid) were enclosed in a water sphere of diameter 40 Å, and the steroid position and orientation were weakly restrained by applying harmonic positional restraints (5 kcal/mol/Å^2^) to three carbon atoms of the ring system. To account for the 55 M standard state, a 10 kcal/mol/Å^2^ distance restraint was applied between the acetate acceptor oxygen and the steroid donor carbon, which keeps these atoms in contact during the simulations. A soft exponential repulsion term (*U*_rep_ = *A*e^–β*r*_*ij*_^) was applied between the donor and acceptor atoms with *A* = 12 100 kcal/mol and β = 2.5 Å^–1^. At each temperature (290, 295, 300, 305, and 310 K), 60 replicate MD/EVB free energy calculations were carried out with a 1 fs time step. Each of these replicas involved 200 ps of initial equilibration, followed by 1.05 ns of data collection during the FEP transformation, which involved 21 discrete λ windows. This gives error bars (s.e.m.) for the calculated activation and reaction free energies of less than 0.1 kcal/mol at all temperatures. The MD/EVB simulations (also for the Na + Na^+^ system) were performed with the Q software package,^21^ where water molecules at the sphere boundary were subjected to radial and polarization restraints following the SCAAS model.^21,29^ Water–water Lennard-Jones interactions were truncated beyond 10 Å and long-range electrostatic interactions beyond this cutoff were treated with the local reaction field multipole expansion method.^30^

### Simulation the HBDH Reaction

The HBDH calculations presented are taken from ref (23), where details of the simulations are given. Briefly, they were performed using the GROMACS 2022 software^22^ with the OPLS-AA/L force field^31^ and utilized the crystallographic structure with PDB code 6ZZP(11) and the starting point. The protonation states of the ionizable residues were calculated with PROPKA,^32^ and the system was enclosed in a dodecahedral box with its nearest wall at 20 Å from the edge of the protein. The box encapsulates 23 710 water molecules and nine Na^+^ ions, for a total number of 75 191 atoms. The topologies for the MD/EVB simulations were generated using the gmxtools presented in ref (23) since, for each FEP window, a separate topology corresponding to the appropriate λ needs to be provided.^23^ The system was initially equilibrated in the reactant configuration in the NPT ensemble for 70 ns at 283 K. Following an additional short equilibration at the different target temperatures, 20 replicate FEP calculations were carried out with 51 λ windows for a total simulation time of 20.04 ns at each temperature. The Arrhenius plots were obtained from simulations at 273, 283, 293, 303, and 313 K. During the FEP simulations, coordinates were recorded every 10 time steps and were later used to recalculate the energies corresponding to the main diagonal of the EVB matrix. This was accomplished using the rerun option of the mdrun tool of GROMACS. The energies for each of the two valence states were then collected with the energy tool of GROMACS and analyzed with a modified version^23^ of the Qfep tool of the Q software.
